# Simulation of Biomass Yield and Soil Organic Carbon under Bioenergy Sorghum Production

**DOI:** 10.1371/journal.pone.0115598

**Published:** 2014-12-22

**Authors:** Fugen Dou, Jason P. Wight, Lloyd T. Wilson, Joseph O. Storlien, Frank M. Hons

**Affiliations:** 1 Texas A&M AgriLife Research & Extension Center at Beaumont, Beaumont, Texas, United States of America; 2 Department of Soil and Crop Sciences, Texas A&M University, College Station, Texas, United States of America; Agricultural Research Service, United States of America

## Abstract

Developing sustainable management practices including appropriate residue removal and nitrogen (N) fertilization for bioenergy sorghum is critical. However, the effects of residue removal and N fertilization associated with bioenergy sorghum production on soil organic carbon (SOC) are less studied compared to other crops. The objective of our research was to assess the impacts of residue removal and N fertilization on biomass yield and SOC under biomass sorghum production. Field measurements were used to calibrate the DNDC model, then verified the model by comparing simulated results with measured results using the field management practices as agronomic inputs. Both residue removal and N fertilization affected bioenergy sorghum yields in some years. The average measured SOC at 0–50 cm across the treatments and the time-frame ranged from 47.5 to 78.7 Mg C ha^−1^, while the simulated SOC was from 56.3 to 67.3 Mg C ha^−1^. The high correlation coefficients (0.65 to 0.99) and low root mean square error (3 to 18) between measured and simulated values indicate the DNDC model accurately simulated the effects of residue removal with N fertilization on bioenergy sorghum production and SOC. The model predictions revealed that there is, in the long term, a trend for higher SOC under bioenergy sorghum production regardless of residue management.

## Introduction

Changes in cropping systems affect the amount of plant residue returned to the soil and thus may affect soil organic carbon (SOC) dynamics. Although manure or biosolids are applied to croplands in some areas, crop residues are the main above- and belowground sources of organic C for the SOC pool [1]–[3]. One bioenergy crop production strategy is to harvest or remove the aboveground biomass as much as possible. Such management practices may have undesired impacts including decreased SOC [4]–[5], soil structure [6]–[7], soil microbial activities and biodiversity, and soil fertility [8], and increased soil erosion [9]–[10]. Reduction in SOC is a major concern since one main motivation of lignocellulose (second generation bioenergy crops) production is to reduce carbon dioxide (CO_2_) emissions. In addition, SOC is an ecological driver of soil quality and sustainability. For example, many studies have indicated that changes in SOC impact soil physical [11]–[12], chemical [13]–[14], and biological properties [15]–[16].

To achieve the yield potential of bioenergy crops, many management practices have been tested, including using different cropping systems, tillage methods, and nutrient applications, which may affect SOC differently. The effects of cropping systems on SOC have been broadly studied in conventional crop production. For example, *Dou et al.* [17] and *Dou* [18] reported that increased nitrogen (N) fertilization and diversified cropping systems significantly increased SOC in a 20-yr field experimental trial. Similar results have been reported by *Dou* [19] and Chirinda [20]. These observations have been generalized by *West et al.* [21] in a global survey, indicating that crop rotation can enhance SOC sequestration in surface soils in most cases. The effects of tillage on SOC had been well investigated [22]. The basic underlying mechanism is that conservation tillage reduces soil aggregation, especially macroaggregtate turnover, and thus enhances the protection of microaggregate and associated SOC [23]. This theory has been used well to explain increased SOC under no till or conservation till.

To maintain or increase SOC, optimal management practices including residue return are proposed. Although the main goal of bioenergy crop production is to maximize economic returns, this does not necessarily equate to maximizing harvested biomass. Appropriate residue management is critical to bioenergy crop production along with soil C sequestration. In conventional row crop production, residue return or removal has different impacts on SOC. For instance, *Duiker et al.* [24] reported that an increase in SOC had a linear relation with the amount of wheat residue returned to the soil in a 7-yr field trial in Ohio. Similar results have been summarized in a review by *Follett* [25]. However, not all studies have reported positive effects of residue return on SOC [26]. Such information is quite limited in bioenergy sorghum crop studies. *Wight* [27] reported that 25% residue removal increased the yield of bioenergy sorghum production in College Station, TX but did not affect the yield in Weslaco, TX in 2008. Soil organic C decreases with excessive corn stoves removal regardless of tillage or cropping system [28].

The effects of residue removal on SOC may be compounded by other factors. For example, soil has a limited capacity to store organic C [29]–[30]. In other words, SOC will be saturated or reach an equilibrium for an agroecosystem. The capacity of a soil to sequester additional SOC depends heavily on the initial status of the SOC. Even though a soil has a high capability to store extra C, such changes in SOC may not be achieved in the beginning of land use change. One reason is that total SOC is a larger pool than the annual C additions due to residue return, making annual changes difficult to detect against background levels. Also, some studies have reported that SOC usually does not increase or can even decreases immediately following implementation of conservation tillage to manage C sequestration in conventional crop production [21]. Many efforts have been made to develop sensitive C indexes including soil microbial biomass C, particulate organic C, mineralizable C, and extractable organic C [31] to track possible changes in SOC. However, such measurements may not be helpful in providing information regarding long-term changes in SOC.

Process-based biogeochemical models have been developed to partially fulfill the need for long-term management decisions. A process-based model mainly focuses on simulating the effects of soil physical, chemical, and biological processes on nutrients (carbon, nitrogen, etc.) and primary production which researchers are interested. For example, changes in SOC have been reasonably predicted for different conventional cropping systems using the DeNitrification-DeComposition (DNDC) model [32]–[33]. Other biogeochemistry models, including RothC, CANDY, CENTURY, DAISY and NCSOIL, make similar predictions in SOC changes [34]. For example, *Smith* [35] reported that using Century, DayCentury, DNDC, and Campbell models, crop residue removal significantly affected SOC in wheat and other row crop production systems in Canada and Northern USA.

The overall goal of this study was to evaluate the effect of residue return and N fertilization on biomass yield and SOC under bioenergy sorghum production by using the DNDC model. The DNDC model has been used to estimate residue removal effects on SOC in North America [35], crop production and management effects on SOC in China [36]–[38], and the effects of forest and cropland on SOC in Europe [39]–[40]. We parameterized and validated the DNDC model using a five-yr field trial and predicted the long-term impacts of aboveground residual return on SOC.

## Materials and Methods

### Field Experiment and Soil and Biomass Sampling

Field studies for bioenergy sorghum production were first established at the Texas A&M AgriLife Research Farm near College Station, TX (30.32^o^ N, 94.26^o^ W) in 2008. This region has a mean annual temperature of 20°C and averages 978 mm of annual precipitation. Soil at the site was classified as a Weswood silty loam (fine, mixed, thermic Udifluventic Ustochrept). The soil has a pH of 8.2 (1∶2 soil/water) and an organic C concentration of 0.8 g C kg^−1^ soil. Prior to the study, the field was in cotton production in 2007, and rotated annually with corn. The field was under conventional disk tillage.

A completely randomized block design with four replications was used to test the effects of two factors, residue return and N fertilization, on SOC. The whole field strip was evenly divided into four blocks. Each block had six plots randomly assigned to one of each treatment combination. Plots were 9.14-m long by 4.08-m wide with four rows. Bioenergy sorghum was planted in 2008, 2009, 2010, 2011, and 2012. A severe lodging was observed in 2010, so the residue was manually harvested and returned by the same machine as was being used in the other years. Residue return rates were 0, 25 or 50% of sorghum biomass yield after harvest. Nitrogen was applied to sorghum at either 0 (without N fertilization) or a sufficient rate (with N fertilization); 300 kg N ha^−1^ in 2008 and 250 kg N ha^−1^ thereafter. Nitrogen as urea was sidedress applied 15-cm deep approximately 6 weeks after planting which was at the 4- to 5-leaf stage for sorghum. The bioenergy sorghum used in this study was “4-Ever Green”, a modern photoperiod-sensitive, one-cross hybrid with high biomass yield and low lodging potentials (Walter Moss Seed Co, Waco, Texas, U.S.A.). Planting dates ranged from late March to late April, with a seeding rate of 160,000 ha^-1^. Bioenergy sorghum was managed under conventional tillage. After the final harvest each year, plots were disked twice times to a depth of 15–20 cm, and bedded. Furrow irrigation was minimally performed as needed to maintain sorghum health and dates and amounts are given in **Table 1**.

**Table 1:** Field operations and dates performed at College Station, Texas.

Operation	2008	2009	2010	2011	2012
Pre-plant herbicide application	3rd March	9th March	17th March	2nd March	19th March
Soil sampling	24th March	6th April	17th March	14th March	7th March
Pre-plant disking	25th March	6th April	17th March	24th March	16th March
Planting	26th March	7th April	13th April	25th March	19th March
Interrow cultivation	24th April	5th June	22th May	5th June	25th May
Fertilization	1st May	20th June	22th May	5th May	26th April
Irrigation	10th June 9 cm	16th July 6 cm	31th May 9 cm	12th April	n/a
	10th June 9 cm	24th August 6 cm	n/a	n/a	n/a
Harvest*	5th August	5th November	7th October	1st September	13th August
	14th October	n/a	n/a	n/a	n/a
Bedding	15th October	6th November	12th October	5th September	27th March

* Only for 2008, we harvested twice to test the different harvesting effect on sorghum biomass yield.

n/a refers to data not available.

Composite soil samples with three cores were collected each spring prior to planting sorghum at depth increments of 0–5, 5–15, 15–30, 30–60, and 60–90 cm. However, only soil data at the depth of 0–50 cm was used because the DNDC model simulates SOC changes at this depth. Soil samples were oven dried at 105°C and finely ground to measure SOC by combustion using an Elementar Americas Inc, VarioMAX CN analyzer (Mt. Laurel, NJ, U.S.A.).

Biomass samples and yields were measured by harvesting the two inner rows of each four-row plot. Water content of aerial plant biomass from each plot was determined by taking a random subsample of the chopped plant material after it had passed through the harvester-chopping machine. An approximately 600-g subsample was weighed, oven dried at 60°C for 7 d, and then re-weighed to determine water content. The biomass C and N content were analyzed using the same procedures as for the soil samples. More detailed information on the field setup and sampling can be found in *Wight et al.* [27].

### DeNitrification–DeComposition (DNDC) Modeling

The DNDC model consisted of six submodels that are divided into two primary components [35]. This model simulates C and N cycling in agroecosystems at a daily and hourly time-step. Soil/climate, crop growth, and organic matter decomposition are primary submodels, with nitrification, denitrification and fermentation as secondary submodels [41]. The soil/climate submodel allows for estimation of soil temperature and water profiles, soil water flow and soil water uptake by plants on an hourly basis. The crop growth submodel simulates growth of various crops, predicting plant biomass and N content of grain, stalk and roots. Crop growth is limited by nitrogen and water availability to roots. The decomposition submodel has four C pools: litter, soil microbial biomass, humad (labile humus) and passive humus, with each pool having a fixed decomposition rate and a fixed C:N ratio. Decomposition is influenced by soil texture, water, temperature and nitrogen limitations. The C:N ratio of the bulk soil will change based on the fraction of SOC in litter, soil microbial biomass, humad and humus pools. The pool sizes will change depending on the quantity of organic C from crop residue that enters the soil and rates of decomposition, which are determined primarily by soil climate, properties, and cultivation. The nitrification and denitrification submodels operate on an hourly time-step and are regulated through an anaerobic balloon concept [42] based on soil water, temperature and redox potential.

### Model Input

To drive the DNDC model, climate, soil, crop parameters, and management practices data are required. The climate data for this location was obtained from the National Climate Data Center of National Oceanic and Atmospheric Administration (ID: GHCND:USW00003904 with 30.58917°, −96.36472°). The soil information including pH, soil texture, SOC, clay fraction, and water holding capacity was measured. The data on soil wilting point, porosity, and hydraulic conductivity was collected from USDA soil survey publications (www.nrcs.usda.gov). Specific SOC turnover rates, SOC pool partitioning, and other soil input parameters were adapted from the default values. The parameters for soil organic C profile of the DNDC model were adjusted according to field measurement. Management practices were taken from the field operation records (**Table 1**). Crop parameters were obtained by analyzing harvested plant tissues and a literature review on bioenergy sorghum. Briefly, the C:N ratios for the biomass sorghum leaf, stem, and root were 34, 51, and 49 by measuring the C and N content of each component at maturity, respectively. The crop residue return was manually adjusted to reflect the corresponding field operation.

### Model Calibration and Verification

Prior to model application, we calibrated the DNDC model against the results of a randomly selected field treatment. The measurements used for model calibration included dry biomass yield and SOC. Three statistics were used to assess the goodness of fit, including the correlation coefficients (r), the root mean square error (RMSE), and relative error (E). The correlation coefficient is useful in assessing how well the shape of the simulation matches the shape of the measurement [34].

The bias in the total difference between simulation and measurement was determined by calculating the RMSE (equation 1) and E (equation 2) [34]. 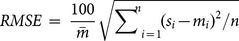
(1)


Where, 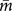, n, i, s_i_, and m_i_ represent mean of the measurement, number of pairs, the *i*th simulation or measurement of the n, the *i*th simulation, and the *i*th measurement. Smaller RMSE or E value indicates more accurate simulation. 
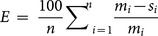
(2)


To project the long-term effect of residue removal on SOC, we generated a series of 50-yr projections using the DNDC model beginning at the end of the experiment observation period because SOC usually establishes a new equilibrium due to changes in management practices [21]. The starting point for all simulations was the SOC with optimal N fertilization for sorghum (250 kg N ha^−1^) without residue return during 2012. All other management practices including fertilization, planting and harvesting dates, and cultivation were also the same as for year 2012. The weather data, including maximum and minimum temperatures and precipitation, were from 1952 to 2013 from the National Climate Data Center of NOAA (2013) but were randomly arranged through a Monte-Carlo simulation [43].

### Model Calibration

The bioenergy sorghum treatment with 25% residue return and without N fertilization was randomly chosen to calibrate the model by following the step by step instructions for DNDC model calibration [44]. The correlation coefficients for dry biomass yields and SOC were 0.97 and 0.98 (**Table 2**), respectively. The small RMSE and E values (most less than 18%) for dry biomass yields and SOC indicated a small bias and suggests DNDC accurately predicts the biomass production and changes in SOC during the experiment.

**Table 2:** Statistical tests applied for agreement between simulated and measured values of biomass yield and soil organic C (SOC) under bioenergy sorghum production at College Station, Texas.

Management Practices		Correlation	
N	Residue Return Rate (%)	Biomass	SOC
-N	0	0.65	0.96
-N	25	0.97	0.98
-N	50	0.96	0.98
+N	0	0.74	0.88
+N	25	0.95	0.91
+N	50	0.90	0.99

		Root of Mean Square Error	
-N	0	13	13
-N	25	16	7
-N	50	15	13
+N	0	18	3
+N	25	14	10
+N	50	9	7

		Relative Error	
-N	0	17	16
-N	25	27	8
-N	50	12	−12
+N	0	−12	2
+N	25	16	13
+N	50	−4	0

The nitrogen (N) rate was 300 kg N ha^−1^ for 2008 and 250 kg N ha^−1^ thereafter.

### Statistical Analysis

The field observation data were analyzed with SAS (SAS 9.2, 2009). A PROC MIXED procedure was used for individual treatment comparisons at *P*<0.05. Year was used as a repeated measure variable. We also used type  =  hf and un but both did not meet the criteria of convergence.

## Results and Discussion

### Effects of residue removal and nitrogen fertilization on bioenergy sorghum yield

Our measured results indicated that residue removal affected bioenergy sorghum yield in some years (**Tables 3** **and** **4**). For example, in 2011 and 2012, 25% residue return had greater biomass yield than other treatments. Smith [35] also reported that residue removal decreased grain yields for winter wheat, barley, and corn in Canada. The possible reason may be because residue return can affect soil moisture and/or nutrient supply. Such yield decreases may indicate there was an optimal rate for residue return to approach the yield potential of bioenergy sorghum.

**Table 3:** Analysis of variance for biomass yield as affected by N fertilization, residue return, and their interaction in 2008, 2009, 2010, 2011, and 2012.

Source	DF	Pr>F
N Fertilization (N)	1	<.0001
Residue (R)	2	0.0003
R*N	2	0.1424
Year (Y)	4	<.0001
N*Y	4	0.5043
R*Y	8	0.2942
N*R*Y	8	0.7238

DF and Pr refer to degree of freedom and probability, respectively.

**Table 4:** Measured and simulated dry biomass yields (Mg ha^−1^) as affected by residue removal and N fertilization under bioenergy sorghum at College Station, TX.

N Fertilization (kg N ha^−1^)	Residue Return (%)	Measured Biomass (Mg ha^−1^)	Simulated Biomass (Mg ha^−1^)
----------------2008----------------			
-N*	0	(17.8±4.8†)b‡	18.7
-N	25	(22.4±4.2)ab	18.7
-N	50	(20.6±1.6)ab	18.7
+N	0	(22.4±3.7)ab	26.3
+N	25	(24.4±3.2)a	26.3
+N	50	(22.6±2.5)ab	26.3
----------------2009----------------			
-N	0	(13.7±1.7)c	10.2
-N	25	(14.2±3.9)bc	10.1
-N	50	(14.4±5.3)bc	10.2
+N	0	(19.7±1.9)ab	16.4
+N	25	(22.6±4.6)a	16.8
+N	50	(17.2±4.7)abc	17.1
----------------2011----------------			
-N	0	(11.7±1.5)c	10.0
-N	25	(12.7±2.3)c	10.4
-N	50	(10.9±2.7)c	10.9
+N	0	(12.4±3.1)c	17.7
+N	25	(23.0±3.8)a	18.6
+N	50	(18.9±1.7)b	19.7
----------------2012----------------			
-N	0	(20.6±1.2)bc	13.5
-N	25	(23.6±3.4)bc	13.4
-N	50	(15.0±3.2)c	13.7
+N	0	(24.6±7.4)b	25.4
+N	25	(35.0±11.1)a	25.6
+N	50	(26.7±4.7)ab	25.9

* -N and +N refer to without N fertilization and with a N rate at 300 kg N ha^-1^ for 2008 and 250 kg N ha^−1^ thereafter.

† means with standard deviation.

‡ Mean values within the same year labeled with different letters differ significantly at P<0.05.

Unlike residue removal, N application consistently increased biomass yield across all three residue removal treatments in 2009, 2011, and 2012 (**Table 3**), indicating that the native soil N was not sufficient to support high yield of bioenergy sorghum. Similar results have been reported by Erickson [45]. Maughan [46] observed that energy sorghum responded to increased N up to 224 kg N ha^−1^, achieving biomass yield of 35.1 Mg ha^−1^. Although bioenergy sorghum has a lower C:N ratio of the aboveground biomass than most cereal crops, the overall high biomass means a large demand for N. The study of Wight [27] indicated that aboveground bioenergy sorghum could contain up to 254 kg N ha^−1^, depending on the yield. If the belowground biomass was taken into account, the overall demand for N would be greater. The same pattern was also observed for the simulated results. Such consistence in N effects on biomass yield suggested that DNDC model reasonably simulated the response of bioenergy sorghum crop response to N application. The correlation coefficient between measured and simulated biomass yield ranged from 0.65 to 0.97 (**Table 2**) indicating that the calibrated DNDC model simulated most of the effects of residue removal and N fertilization on bioenergy sorghum production.

### Effects of residue removal and nitrogen fertilization on SOC under bioenergy sorghum

**Figure 1:**
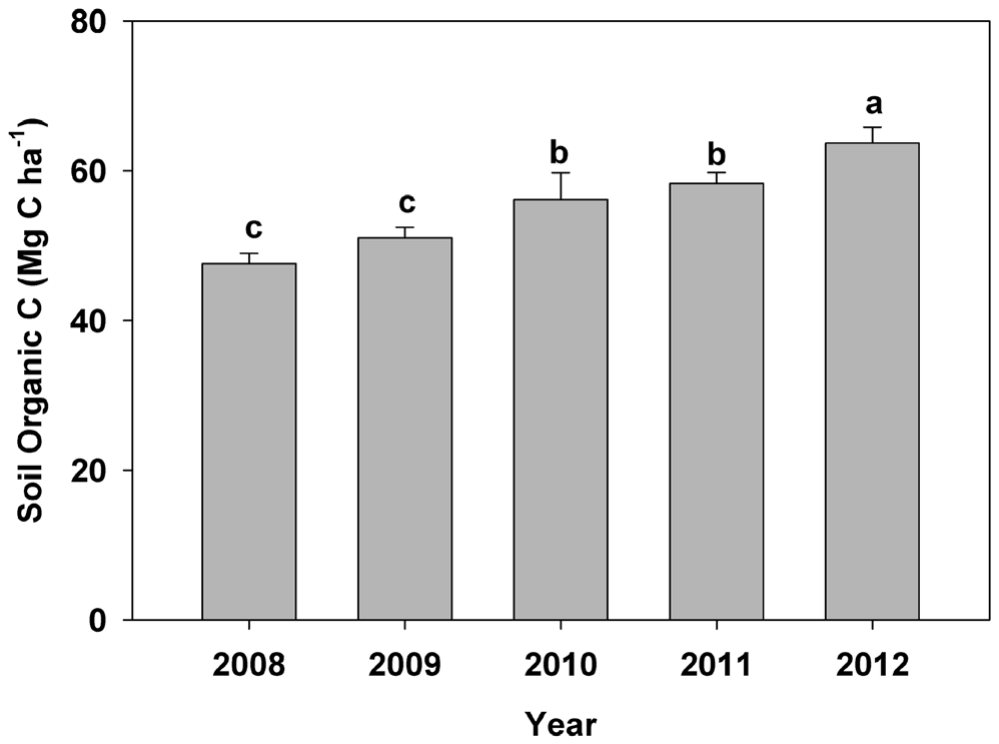
Soil organic C variation with year in the upper 50 cm at College Station, Texas.

The measured SOC data indicated that both residue removal and N fertilization did not significantly affect SOC (**Table 5**). Without N fertilization, increased residue removal numerically increased SOC (**Table 6**) in 2011, which was inconsistent with our hypothesis. We would expect an increase in SOC with decreasing residue removal. Similar results were reported by *Smith* [35]. The possible reason has not been well developed. However, SOC from all treatments had a temporal pattern in that SOC increased with time following implementation of the production practice (**Fig. 1**). Crop residue is the main source of C for SOC. Many studies have reported that increased SOC is usually expected with increased incorporation of crop residue [6], [47], [7]. *Blanco-Canqui et al.* [4] reported that SOC under 80% residue return was significantly greater than that under complete residue removal in a long-term field trial. Thus the different effect of residue return on SOC between the discussed studies may indicate that root biomass plays a significant role in C sequestration in our study.

**Table 5:** Analysis of variance for soil organic C as affected by N fertilization, residue return, and their interaction in 2008, 2009, 2010, 2011, and 2012.

Source	DF	Pr>F
N Fertilization (N)	1	0.8172
Residue (R)	2	0.0102
R*N	2	0.0487
Year (Y)	4	0.0004
N*Y	4	0.9949
R*Y	8	0.9056
N*R*Y	8	0.2882

DF and Pr refer to degree of freedom and probability, respectively.

**Table 6:** Measured and simulated soil organic C (Mg C ha^−1^) as affected by residue removal and N fertilization under bioenergy sorghum at College Station, Texas.

N Fertilization (kg N ha^−1^)	Residue Return (%)	Measured SOC (Mg C ha^−1^)	Simulated SOC (Mg C ha^−1^)
----------------2008----------------			
-N*	0	(57.9±6.4†)ab‡	56.3
-N	25	(57.5±12.3)ab	57.4
-N	50	(47.5±5.5)b	59.2
+N	0	(52.9±9.0)ab	56.6
+N	25	(62.4±12.7)a	58.0
+N	50	(52.9±7.2)ab	60.1
----------------2009----------------			
-N	0	(66.5±6.5)	56.6
-N	25	(57.7±13.6)	58.1
-N	50	(53.1±7.3)	60.5
+N	0	(55.9±10.1)	57.6
+N	25	(64.0±10.1)	59.6
+N	50	(60.6±11.3)	62.9
----------------2010----------------			
-N	0	(71.8±9.2)ab	57.6
-N	25	(57.6±16.4)ab	58.8
-N	50	(55.0±6.2)b	60.9
+N	0	(57.8±8.6)ab	59.5
+N	25	(66.2±8.4)ab	61.1
+N	50	(85.1±50.1)a	63.9
----------------2011----------------			
-N	0	(72.3±5.4)ab	58.0
-N	25	(68.2±13.6)abc	59.7
-N	50	(57.6±2.6)c	62.7
+N	0	(60.6±4.5)bc	59.6
+N	25	(74.7±6.9)a	62.1
+N	50	(70.9±10.8)ab	66.4
----------------20012----------------			
-N	0	(78.7±18.3)	58.7
-N	25	(76.0±15.7)	60.4
-N	50	(62.9±11.2)	63.3
+N	0	(72.7±5.9)	60.6
+N	25	(78.6±22.4)	63.0
+N	50	(77.5±11.6)	67.3

*-N and +N refer to without N fertilization and with a N rate at 300 kg N ha^−1^ for 2008 and 250 kg N ha^−1^ thereafter.

† means with standard deviation.

‡ Mean values within the same year labeled with different letters differ significantly at P<0.05.

Although N fertilization increased biomass production, SOC was not significantly affected by N fertilization (**Tables 5** **and** **6**). Increased N supply through fertilization has been reported to stimulate SOC turnover and may decrease SOC compared to treatments without N [48]. However, the effect of N fertilization on SOC in this study was in contrast with the results reported by Dou and Hons [17] from a similar site which has been implemented for more than 20 years. Those authors reported that N fertilization increased SOC in a wheat cropping system. During the transition of cropping systems change, SOC may have a different response to management practices including N fertilization along with environmental conditions.

When all simulated SOC data were pooled together, the correlation between simulated and observed data did not give a good fit. However, with N fertilization only, the correlation was high (R^2^>0.8), indicating that the DNDC model reasonably well simulated the effect of residue removal on SOC under biomass sorghum production. The simulated SOC at 0–50 cm which ranged from 56.3 to 67.3 Mg C ha^−1^ (**Table 6**) was within the range of measured SOC (47.5 to 85.1 Mg C ha^−1^) at the same depth.

### Long-term effects of residue removal on SOC under bioenergy sorghum

**Figure 2:**
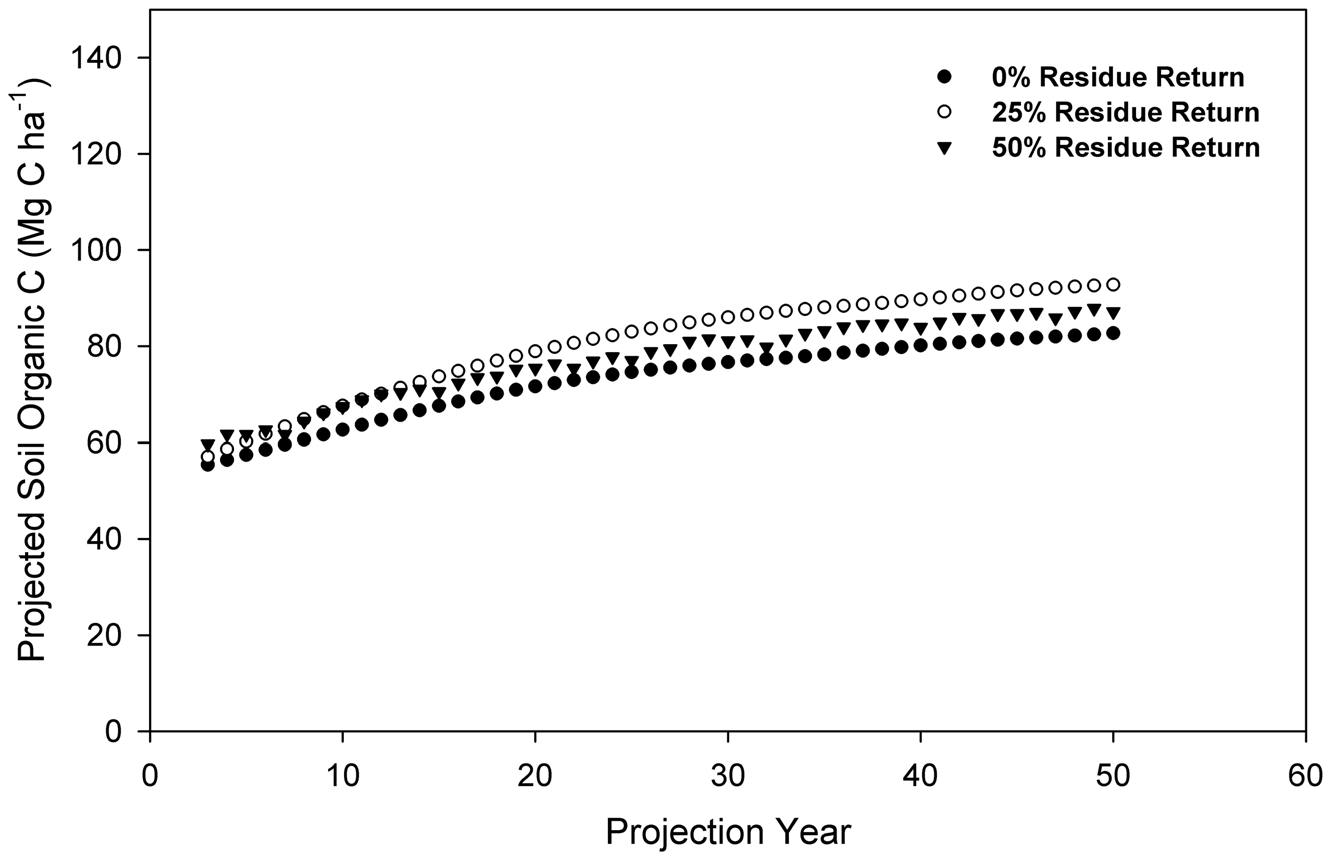
Simulated soil organic C in a 50-yr period for the upper 50 cm of soil as affected by different residue removal rates under optimal N fertilization at College Station, Texas.

Simulated SOC increased for each of the treatments over time (**Fig. 2**) regardless of the rate of residue removal. Generally, the rate of SOC increase slightly decreased over time, suggesting the establishment of a new equilibrium. Compared with the 0% residue return, partial residue return (25% or 50%) has greater SOC, especially for the 25% residue return. Our results contrasted with those reported by *Galdos et al.* [49], which suggests that SOC under sugar cane with residue burning should decrease with time. The differences in SOC projection may be due to the difference in crop species or the approach of residue removal. Burning may cause extra loss of SOC. In addition, bioenergy sorghum may have greater residual C input than sugar cane due to sorghum being an annual crop with all of its roots dying following harvest each year.

## Conclusions

We assessed the impact of residue removal and N fertilization on biomass yield and SOC to a depth of 50 cm in south central USA based on results from a system of 5-yr field trials. Our field observations indicated that biomass yield could be improved with N fertilization. The effect of residual removal on biomass yield depended on the N rate and sampling year. For SOC, the measured data indicated that both residue removal and N fertilization did not significantly affect SOC for most of treatments. Moreover, we used the DNDC model to project the long-term (50-yr) effects of residue management on SOC. The high correlation coefficients between measured and simulated SOC indicated that the DNDC model simulated the major effects of residue return with N fertilization on SOC. A similar result was also observed for biomass yield. Our long-term (50-yr) projection also suggests that SOC under different residue removal treatments increased with time. These results suggest that our current management practices for bioenergy sorghum will increase SOC sequestration.

## Supporting Information

S1 Data (XLSX)

## References

[1] Lal R (2004) Soil carbon sequestration to mitigate climate change. Geoderma 123:1–22.

[2] Rasse DP, Rumpel C, Dignac MF (2005) Is soil carbon mostly root carbon? Mechanisms for a specific stabilisation. Plant Soil 269:341–356.

[3] Wilhelm WW, Johnson JMF, Hatfield JL, Voorhees WB, Linden DR (2004) Crop and soil productivity response to corn residue removal: A literature review. Agron J 96:1–17.

[4] Blanco-Canqui H, Lal R (2009) Crop residue removal impacts on soil productivity and environmental quality. Cr Rev Plant Sci 28:139–163.

[5] Cibin R, Chaubey I, Engel B (2012) Simulated watershed scale impacts of corn stover removal for biofuel on hydrology and water quality. Hydrol Process 26:1629–1641.

[6] Hammerbeck AL, Stetson SJ, Osborne SL, Schumacher TE, Pikul JL (2012) Corn residue removal impact on soil aggregates in a no-till corn/soybean rotation. Soil Sci Soc Am J 76:1390–1398.

[7] Moebius-Clune BN, van Es HM, Idowu OJ, Schindelbeck RR, Moebius-Clune DJ, et al (2008) Long-term effects of harvesting maize stover and tillage on soil quality. Soil Sci Soc Am J 72:960–969.

[8] Zhao G, Bryan BA, King D, Luo ZK, Wang EL, et al (2013) Impact of agricultural management practices on soil organic carbon: Simulation of Australian wheat systems. Glob Change Biol 19:1585–1597. 10.1111/gcb.12145 23504769

[9] Pedroli B, Elbersen B, Frederiksen P, Grandin U, Heikkila R, et al (2013) Is energy cropping in Europe compatible with biodiversity? - Opportunities and threats to biodiversity from land-based production of biomass for bioenergy purposes. Biomass Bioenergy 55:73–86.

[10] Rufino MC, Dury J, Tittonell P, van Wijk MT, Herrero M, et al (2011) Competing use of organic resources, village-level interactions between farm types and climate variability in a communal area of NE Zimbabwe. Agr Sys 104:175–190.

[11] Baker JM, Ochsner TE, Venterea RT, Griffis TJ (2007) Tillage and soil carbon sequestration - What do we really know? Agr Ecosyst Environ 118:1–5.

[12] Balesdent J, Chenu C, Balabane M (2000) Relationship of soil organic matter dynamics to physical protection and tillage. Soil Till Res 53:215–230.

[13] Guo LB, Gifford RM (2002) Soil carbon stocks and land use change: A meta analysis. Glob Change Biol 8:345–360.

[14] Reeves DW (1997) The role of soil organic matter in maintaining soil quality in continuous cropping systems. Soil Till Res 43:131–167.

[15] Cambardella CA, Elliott ET (1994) Carbon and nitrogen dynamics of soil organic-matter fractions from cultivated grassland soils. Soil Sci Soc Am J 58:123–130.

[16] Cambardella CA, Moorman TB, Novak JM, Parkin TB, Karlen DL, et al (1994) Field-scale variability of soil properties in central Iowa soils. Soil Sci Soc Am J 58:1501–1511.

[17] Dou F, Hons FM (2006) Tillage and nitrogen effects on soil organic matter fractions in wheat-based systems. Soil Sci Soc Am J 70:1896–1905.

[18] Dou F, Wright AL, Hons FM (2008) Sensitivity of labile soil organic carbon to tillage in wheat-based cropping systems. Soil Sci Soc Am J 72:1445–1453.

[19] Dou F, Hons FM, Ocumpaugh WR, Read JC, Hussey MA (2013) Soil organic carbon pools under switchgrass grown as a bioenergy crop compared to other conventional crops. Pedosphere 23:409–416.

[20] Chirinda N, Olesen JE, Porter JR (2012) Root carbon input in organic and inorganic fertilizer-based systems. Plant Soil 359:321–333.

[21] West TO, Post W (2002) Soil organic carbon sequestration rates by tillage and crop rotation: A global data analysis. Soil Sci Soc Am J 66:1930–1946.

[22] Oades JM (1984) Soil organic matter and structural stability: mechanisms and implications for management. Plant Soil 76:319–337.

[23] Six J, Elliot E, Paustian K (2000) Soil macroaggregate turnover and microaggregate formation: a mechanism for C sequestration under no-tillage agriculture. Soil Biol Biochem 32:2099–2103.

[24] Duiker SW, Lal R (1999) Crop residue and tillage effects on carbon sequestration in a Luvisol in central Ohio. Soil Till Res 52:73–81.

[25] Follett RF (2001) Soil management concepts and carbon sequestration zin cropland soils. Soil Till Res 61:77–92.

[26] Solberg ED, Nyborg M, Izaurralde RC, Malhi SS, Janzen HH, et al. (1998) Carbon storage in soils under continuous cereal grain cropping: N fertilizer and straw, In: Lal, R., Kimble, J.M., Stewart, B.A., Follett, R.F. (Eds.), Management of carbon sequestration in soil. Boca Raton, FL.: CRC Press. pp. 235–253.

[27] Wight JP, Hons FM, Storlien JO, Provin TL, Shahandeh H, et al (2012) Management effects on bioenergy sorghum growth, yield and nutrient uptake. Biomass Bioenergy 46:593–604.

[28] Blanco-Canqui H (2013) Crop residue removal for bioenergy reduces soil carbon pools: How can we offset carbon losses? Bioenergy Research 6:358–371.

[29] Six J, Conant RT, Paul EA, Paustian K (2002) Stabilization mechanisms of soil organic matter: Implications for C-saturation of soils. Plant Soil 241:155–176.

[30] Stewart CE, Paustian K, Conant RT, Plante AF, Six J (2007) Soil carbon saturation: Concept, evidence and evaluation. Biogeochemistry 86:19–31.

[31] Dou F, Wright AL, Hons FM (2008) Dissolved and soil organic carbon after long-term conventional and no-tillage sorghum cropping. Commun Soil Sci Plant 39:667–679.

[32] Li C, Frolking S, Crocker GJ, Grace PR, Klír J, et al (1997) Simulating trends in soil organic carbon in long-term experiments using the DNDC model. Geoderma 81:45–60.

[33] Ouyang W, Qi SS, Hao FH, Wang XL, Shan YS, et al (2013) Impact of crop patterns and cultivation on carbon sequestration and global warming potential in an agricultural freeze zone. Ecol Model 252:228–237.

[34] Smith P, Smith JU, Powlson DS, McGill WB, Arah JRM (1997) A comparison of the performance of nine soil organic matter models using datasets from seven long-term experiments. Geoderma 81:153–225.

[35] Smith WN, Grant BB, Campbell CA, McConkey BG, Desjardins RL, et al (2012) Crop residue removal effects on soil carbon: Measured and inter-model comparisons. Agr Ecosyst Environ 161:27–38.

[36] Tang H, Qiu J, Van Ranst E, Li C (2006) Estimations of soil organic carbon storage in cropland of China based on DNDC model. Geoderma 134:200–206.

[37] Wang L, Qiu J, Tang H, Li H, Li C, et al (2008) Modelling soil organic carbon dynamics in the major agricultural regions of China. Geoderma 147:47–55.

[38] Zhang F, Li C, Wang Z, Wu H (2006) Modeling impacts of management alternatives on soil carbon storage of farmland in Northwest China. Biogeosciences 3:451–466.

[39] Li CS, Farahbakhshazad N, Jaynes DB, Dinnes DL, Salas W, et al (2006) Modeling nitrate leaching with a biogeochemical model modified based on observations in a row-crop field in Iowa. Ecol Model 196:116–130.

[40] Li CS, Mosier A, Wassmann R, Cai ZC, Zheng XH, et al (2004) Modeling greenhouse gas emissions from rice-based production systems: Sensitivity and upscaling. Global Biogeochem Cy 18 doi:10.1029/2003GB002045

[41] Peltoniemi M, Thurig E, Ogle S, Palosuo T, Schrumpf M, et al (2007) Models in country scale carbon accounting of forest soils. Silva Fenn 41:575–602.

[42] Leip A, Marchi G, Koeble R, Kempen M, Britz W, et al (2008) Linking an economic model for European agriculture with a mechanistic model to estimate nitrogen and carbon losses from arable soils in Europe. Biogeosciences 5:73–94.

[43] Erickson JE, Woodard KR, Sollenberger LE (2012) Optimizing sweet sorghum production for biofuel in the southeastern USA through nitrogen fertilization and top removal. Bioenergy Research 5:86–94.

[44] Li C (2012) Steps for calibration and validation of DNDC model. Available: http://www.arb.ca.gov/cc/capandtrade/protocols/rice/steps-for-dndc-12-20-13.pdf. Accessed 2014 Aug.

[45] Maughan M, Voigt T, Parrish A, Bollero G, Rooney W, et al (2012) Forage and energy sorghum responses to nitrogen fertilization in central and southern Illinois. Agron J 104:1032–1040.

[46] Kering MK, Butler TJ, Biermacher JT, Guretzky JA (2012) Biomass yield and nutrient removal rates of perennial grasses under nitrogen fertilization. Bioenergy Research 5:61–70.

[47] Hobbie SE (2005) Contrasting effects of substrate and fertilizer nitrogen on the early stages of litter decomposition. Ecosystems 8:644–656.

[48] Segoli M, De Gryze S, Dou F, Lee J, Post WM, et al (2013) AggModel: A soil organic matter model with measurable pools for use in incubation studies. Ecol Model 263:1–9.

[49] Galdos MV, Cerri CC, Cerri CEP, Paustian K, Van Antwerpen R (2009) Simulation of soil carbon dynamics under sugarcane with the Century model. Soil Sci Soc Am J 73:802–811.

